# Editorial: Beyond the Pharmacology of Psychoactive Plant Medicines and Drugs: Pros and Cons of the Role of Rituals and Set and Setting

**DOI:** 10.3389/fphar.2021.804254

**Published:** 2021-12-03

**Authors:** Alex K. Gearin, Luis E. Luna, Fernando Mendive, Marco Leonti, Claudio Ferrante, Luigi Menghini, Matteo Politi

**Affiliations:** ^1^ Medical Ethics and Humanities Unit, The University of Hong Kong, Hong Kong, China; ^2^ Wasiwaska, Research Center for the Study of Psychointegrator Plants, Visionary Art and Consciousness, Florianópolis, Brazil; ^3^ Takiwasi Center for Rehabilitation of Drug Addicts and Research on Traditional Medicine, Tarapoto, Peru; ^4^ Department of Biomedical Sciences, Faculty of Medicine and Surgery, University of Cagliari, Cagliari, Italy; ^5^ Department of Pharmacy, University “G. d’Annunzio” of Chieti-Pescara, Chieti, Italy

**Keywords:** psychedelic, ritual, ayahuasca, plant medicine, psychoactive drugs

The Research Topic here presented is based on first-hand clinical experiences and ethnographic and observational fieldwork studies on practices used or inspired by traditional medicines related in particular to the so-called psychedelics; these are substances known to trigger non-ordinary states of consciousness, which are currently getting more attention in medicine as reflected by the increase in clinical research and a shift in attitude and opinion among public spheres. A total of 51 potential contributors were contacted, allowing the submission of nine manuscripts, one of which declined the others eight are now published. The controversial class of drugs known as psychedelics derives from a diverse history of shamanic, therapeutic, and recreational uses among peoples across the globe. The broader context has influenced the emerging clinical arts of psychedelic therapy yet also represents a vast realm of alternative approaches. Key to such diversity, the experience of consuming the substances can remarkably absorb social and cultural context and become sharply influenced by parameters of ritual, expectation, and belief. These parameters of psychedelic use mark the focus of this special issue of *Frontiers in Pharmacology*, and their variety among naturalistic settings demonstrate significant differences to the clinical research. We would like to add and highlight one important distinction here. In clinical contexts, psychedelic substances are grammatically assigned an adjunctive role in the common allopathic terminology of “psychedelic-assisted therapies.” The humanistic emphasis on psychedelic substances *assisting* conventional therapies reflects the clinical needs for quality, safety, and efficiency. However, in other social and cultural contexts, psychedelic substances are not necessarily assigned the modest role of assistant. Among alternate contexts to the clinic, as illustrated in several articles in this issue, the grammar is often inverted with the psychedelic substance embodying a transcendent value and being *assisted* by ritual practice and intersubjective encounters. Foregrounding the centrality of special aspects of psychedelic experiences corresponds to indigenous, religious, and spiritual perspectives. Whether placing the substance and psychedelic experiences at the center of the healing process—in the shamanic, religious, and spiritual sense—or confining them to an adjunctive role—in the clinical sense—the different approaches recognize the indispensable role of contextual or “extra-pharmacological” elements in shaping the desired experiences and outcomes of consuming the substances.

With the aim to explore the role of such contextual factors, this special issue provides an array of eight new research and review articles about the cultural diversity of psychedelic plant medicines used in naturalistic settings. In “Relational Processes in Ayahuasca Groups of Palestinians and Israelis,” Roseman et al. examine how the intercultural and intersubjective dynamism of ayahuasca healing ceremonies may promote peacebuilding. Through surveys, interviews, and ethnographic methods aimed at considering the subjective narratives of the ceremonial participants in relation to ritual structure, music, and behavior, the authors illustrate how the psychological process of healing can embody sociopolitical contexts. The classic psychedelic experience reports of mystical unity involved a dissolution of perceived conflicts in national and religious identity. The research marks an important step towards situating psychedelic healing in broader political and historical contexts and reframing suffering and health beyond the level of the individual (patient). In “Psychedelic Communitas: Intersubjective Experience During Group Sessions Predicts Enduring Changes in Psychological Wellbeing and Social Connectedness,” Kettner et al. introduce a revised psychometric scale to investigate the collectivist and psychosocial parameters of psychedelic healing experiences. Through measuring the significance of “communitas,” the results of their observational, web-based survey, suggest that intersubjective parameters of psychedelic use are instrumental to long-term outcomes in positive wellbeing. In “The Evolved Psychology of Psychedelic Set and Setting: Inferences Regarding the Roles of Shamanism and Entheogenic Ecopsychology,” Winkelman undertakes a multidisciplinary approach to investigate the possibility that psychedelic drugs were instrumental to the evolution of hominin (human) cognition and consciousness. Bringing together studies in cross-cultural shamanism, the neuroscience of psychedelic use, and evolutionary psychology, the paper provides an elaborate and provocative investigation into the “Stoned Ape Theory” originally proposed by Terence McKenna, which claims psilocybin mushrooms were an evolutionary catalyst for the emergence of certain higher cognitive faculties. In “Set and Setting in Santo Daime,” Hartogsohn undertakes a detailed analysis of the psychological, social, and cultural factors shaping the use of ayahuasca, known as “daime,” in the Santo Daime tradition, which originated in Brazil but is now practiced across the globe. The article employs ethnographic and textual methods to illustrate how the use of daime is shaped by a rich tapestry of symbolic ques, ritual structures, sensory components, musical dimensions, and social practices, which can be traced to historical and cultural contexts. It helps pioneering a useful foundation for cross-cultural ethnographic research on the importance of “set” and “setting” in psychedelic use. In “The Shipibo Ceremonial Use of Ayahuasca to Promote Well-Being: An Observational Study,” Gonzalez et al. tracked the long-term psychological health of 200 participants who attended a Shipibo ayahuasca healing retreat in remote Peru. The international participants were already healthy or had never been clinically diagnosed with a mental disorder. Indicating the benefits of the intercultural healing center, the prospective study found “a significant increase in psychological well-being, subjective well-being, spiritual well-being, and quality of life” that was sustained for 1 year. In “Influence of Context and Setting on the Mental Health and Wellbeing Outcomes of Ayahuasca Drinkers: Results of a Large International Survey,” Perkins et al. present results from the largest global survey of ayahuasca drinkers. Drawing upon data from 6,877 participants over 40 countries, the research indicates how ceremonial context, therapeutic motivations, and additional supports (such as yoga, tai chi, integration counselling) predicted positive outcomes in wellbeing, self-insight, and mystical experience. The study provides a significant base of evidence for the importance of pairing ayahuasca approaches with additional therapeutic and spiritual modalities. In “Protocol for Outcomes Evaluation of Ayahuasca-Assisted Addiction Treatment: The Case of Takawasi Center,” Rush et al. present a research protocol developed to evaluate the results of a 30-year old intercultural program for substance use disorders that complements Amazonian traditional medicine practices and concepts with modern psychology and medicine. The observational protocol based on mixed methods, quantitatively assesses a range of domains affected in addictions at baseline and follow-up, and explores clients’ and staff’ experiences about the role of set and setting in the rehabilitation processes through semi-structured interviews. The authors aim to shed light on the pathways for recovery from problematic use of psychoactive substances when a psychoactive substance turns out to be, paradoxically, the medicine. In “Amazonian Medicine and the Psychedelic Revival: Considering the ‘Dieta’,” O’Shaughnessy and Berlowitz challenge the notion of psychedelic “set” and “setting” by examining the significance of dietary practices among Indigenous Amazonian ayahuasca healers in Peru. They situate ayahuasca among a wider complex of plant medicine use to highlight the epistemological significance of indigenous healing practices which transcend the clinical dichotomy of the symbolic and the biological.

Overall, the articles in this special issue highlight the relevance of considering social and cultural domains when attempting to explain the benefits of using psychedelic plant medicines. In parallel to such perspectives, the question of mitigating risks involved with psychedelic use would equally benefit from considering social and cultural parameters, as highlighted in some of the research articles above. Social groups and cultural contexts do not simply give meaning to the experiential properties of psychedelic substance use but are necessary ingredients for safely turning such properties into medicine.

